# Acquisition of plasmid-mediated cephalosporinase producing *Enterobacteriaceae* after a travel to the tropics

**DOI:** 10.1371/journal.pone.0206909

**Published:** 2018-12-18

**Authors:** Florian Lorme, Naouale Maataoui, Emilie Rondinaud, Marina Esposito-Farèse, Olivier Clermont, Etienne Ruppe, Guillaume Arlet, Nathalie Genel, the VOYAG-R study group, Sophie Matheron, Antoine Andremont, Laurence Armand-Lefevre

**Affiliations:** 1 AP-HP, Hôpital Bichat, Laboratoire de Bactériologie, Paris, France; 2 INSERM, IAME, UMR 1137 France, Université Paris Diderot, Sorbonne Paris Cité, Paris, France, AP-HP, Hôpital Bichat, URC Paris-Nord, Paris, France; 3 AP-HP, Hôpital Bichat, URC Paris-Nord, Paris, France; 4 INSERM, CIC 1425-EC, UMR1123, Paris, France; 5 AP-HP, Groupe Hospitalier des Hôpitaux Universitaires de l'Est Parisien, Département de Bactériologie, Paris, France; 6 INSERM U1135, CIMI, Team E13, Paris, France, Sorbonne Université, UPMC Université Paris, Paris, France; 7 AP-HP, Hôpital Bichat, Maladies Infectieuses et Tropicales, Paris, France; Institut National de la Recherche Agronomique, FRANCE

## Abstract

Travelers are at high risk of acquiring multi-drug resistant Enterobacteriaceae (MRE) while traveling abroad. Acquisition of extended spectrum beta-lactamase producing Enterobacteriaceae (ESBL-E) while traveling has been extensively described, but not that of plasmid-mediated cephalosporinase producing Enterobacteriaceae (pAmpC-E). Here, we characterized the pAmpC-E acquired in 574 French travelers to tropical areas enrolled in the VOYAG-R study. Among the 526 MRE isolated at return, 57 (10.8%) from 49 travelers were pAmpC-E. The acquisition rate of pAmpC-E was 8.5% (49/574) ranging from 12.8% (25/195) in Asia, 7.6% (14/184) in Latin America to 5.1% (10/195) in Africa. The highest acquisition rates were observed in Peru (21.9%), India (21.4%) and Vietnam (20%). The carriage of pAmpC-E decreased quickly after return with 92.5% of colonized travelers being negative at one month. Most enzymes were CMY types (96.5%, n = 55, only met in *Escherichia coli*), including 40 CMY-2 (70.2%), 12 CMY-42 (21.1%), 1 CMY-6 and two new CMY-2 variants. The remaining were two DHA observed in *Klebsiella pneumoniae*. CMY-2 producing strains were acquired worldwide whereas CMY-42, except for one, were all acquired in Asia. *Bla*_CMY-2_ genes were associated with different plasmid types, including IncI1 (45. 2%), IncF (10%), IncF-IncI (7.5%), IncA/C (5%) and IncR (2.5%) whereas *bla*_CMY-42_ were all associated with IncI1 plasmids. Even though the pAmpC-E acquisition rate was much lower than that of ESBL-E, it was significant, especially in Asia, showing that pAmpC-E, especially CMY-type producing *E*. *coli* have spread in the community settings of tropical regions.

## Introduction

The increasing antibiotic resistance of Enterobacteriaceae is alarming, especially in low and middle incomes countries [1]. It is now well known that travelers are at high risk of acquiring multi-drug resistant Enterobacteriaceae (MRE, *i*.*e*. extended spectrum beta-lactamase [ESBL-E], Plasmid-mediated AmpC [pAmpC-E] and carbapenemases [CPE] producing Enterobacteriaceae) while traveling abroad. Acquisition rates of MRE vary from 20 to 50%, depending on the geographic region of travel and can reach up to 80% after a stay in the Indian subcontinent [2,3]. Digestive disorders, diarrhea and antibiotic use during the trip have been determined as major risk factors associated with the acquisition of MRE [4,5]. Almost all studies assessing the rates and risk factors for MRE acquisition in healthy volunteers have focused on Enterobacteriaceae expressing an ESBL-E [3]. However, resistance to third generation cephalosporins can also be caused by the high-level production of AmpC enzyme expressed, which confers a slightly different phenotype than ESBL enzyme, sparing 4^th^ generation cephalosporin, *i*.*e*. cefepime. This level of expression can be observed in strains carrying over-expressed chromosomal *ampC* following mutational events, or in strains that have acquired a plasmid-encoded *ampC* gene. The first type is mainly associated with antibiotic selection pressure and hospital-acquired infections but the second type has also been described in community-acquired infections [6]. Plasmid-mediated AmpC (pAmpC) genes originate from chromosomal genes that have been mobilized onto plasmids. Based on sequence similarities with species-carrying chromosomal specific AmpC enzymes, pAmpC variants are classified into six phylogenetic groups: ACC from *Hafnia alvei*, DHA from *Morganella morganii*, CMY-2-like/LAT from *Citrobacter freundii*, ACT/MIR from *Enterobacter sp*, FOX from *Aeromonas caviae and* CMY-1/MOX from *Aeromonas hydrophyla*.[7,8]. pAmpC enzymes are not easy to detect phenotypically especially in Enterobacteriaceae species that carry a chromosomal *ampC* gene, as *Escherichia coli*, *Citrobacter freundii*, and *Enterobacter sp*. Consequently, their prevalence is likely underestimated [8]. Yet, their incidence is increasing in a large number of countries [9,10] and their acquisition during travel is largely unknown. Here, we fulfilled this gap in studying their acquisition and persistence after return in a cohort of French travelers to the tropics in whom we had previously described acquisition and persistence of MRE [4].

## Materials and methods

### Strain selection and characterization

This study is an ancillary study of the VOYAG-R clinical trial (clinicaltrials.gov number NCT01526187) in which the acquisition and persistence of MRE was determined in 574 travelers returning from tropical regions [4]. All volunteers who had no detectable MRE fecal carriage before their departure were included in the VOYAG-R study and asked to send a stool sample within a week after their return. Travelers who were carrying MRE after their return were asked to provide stool samples 1, 2, 3, 6, and 12 months later, or until MRE was no longer detected. Fresh stool samples were self-collected at the traveler’s home and promptly shipped to the bacteriology laboratory of Bichat-Claude Bernard Hospital in Paris, France. Each sample was accompanied by a self-completed questionnaire recording (1) at inclusion: demographic data (age, sex), dates of departure and return, the type of travel; and (2) after return: occurrence of diarrhea and/or antibiotic use during the trip. As doxycycline was exclusively used for malaria prophylaxis and not as an antibiotic, we considered it as an antimalarial agent. Follow-up was terminated in case of further travel to a tropical region.

Stool samples were plated on ChromID ESBL agar (bio-Mérieux, Marcy-l’Etoile, France) and bi-valve ESBL agar (AES Chemunex, Ivry-sur-Seine, France). In parallel, an enrichment step was performed by diluting approximately 10 mg of stool in 10 mL of brain-heart infusion broth supplemented with 1.5 mg/L cefotaxime and incubated overnight before plating on ChromID ESBL agar. The antibiotic susceptibility testing (amoxicillin, amoxicillin plus clavulanic acid, cefotaxime, ceftazidime, cefepime, cefoxitine, ofloxacin, gentamicin, amikacin and co-trimoxazole) was performed on all growing colonies by the disk diffusion method as recommended by the European Committee on Antimicrobial Susceptibility Testing [11]. MIC of cefotaxime, ceftazidime and cefepime were determined using E-tests according to the recommendations of the manufacturer (bio-Mérieux, Marcy-l’Etoile, France).

All isolates resistant to cephalosporins had been stored at -80°C. The putative production of AmpC was suspected when isolates were resistant to third generation cephalosporins and/or to cefoxitin with a negative clavulanate-based double disk synergy test. Species harboring a chromosomal *ampC*, except *E*. *coli*, have been excluded of the analysis.

DNA was extracted using the EZ1 DNA tissue kit (Qiagen, Courtaboeuf, France) and PCR tested for the presence of *bla*_CMY-2 like_, *bla*_DHA_, *bla*_MOX_, *bla*_ACC_ genes (S1 Table). All *bla*_CMY-2-like_ genes were sequenced with the Sanger method by the Big Dye Terminator version 1.1 kit in a Applied Biosystems 3130xl Genetic Analyzers (Applied Biosystems, Les Ulis, France).

The phylogenetic group of *E*. *coli* that carried one of these genes was determined as described [12] resulting in their classification into 8 groups (A, B1, B2, C, D, E, F and *Escherichia* cryptic clade I). STc131-O25b *E*. *coli* have been determined using allele-specific PCR, as described [13].

Plasmids were characterized by plasmid replicons and relaxases identification using PRaseT (plasmid relaxase gene typing) and simplex PCR for the IncR and IncJ group as described [14,15].

### Genetic relatedness

Genetic relatedness between pAmpC-E that persisted at one or two month was assessed by repetitive sequence-based PCR (rep-PCR) using the semi-automated Diversilab^TM^ system (BioMérieux, Marcy-l’Étoile, France). The amplified products were separated by electrophoresis on microfluidics chips and analyzed with the Agilent 2100 Bioanalyzer (Agilent Technologies, Palo Alto, CA, USA). The peak and band data were analyzed by DiversiLab^TM^ software (bioMérieux). The cut-off value was 95% for determining genetic similarity.

### Statistical analysis

Multinomial logistic regressions were performed to model the relationship between the predictors and the three groups of acquisition (acquisition of ESBL-E only, acquisition of pAmpC-E, and not acquired any MRE). Statistical significance two-tailed alpha level was set as 0.05% for all tests. All odd ratios presented are relative to the reference category, not acquired any MRE, if not otherwise specified. Analyses were performed using R statistical software v 3.2

### Ethical issues

The VOYAG-R study was approved by the Ile de France IV ethics committee on 14 November 2011. The study was observational and did not directly benefit the participants. According to the French regulation for clinical research, each participant signed a “nonrefusal” form [4].

## Results

### pAmpC-E acquisition rate

Among 63 strains, suspected to produce a plasmid-mediated AmpC, 57 (90.4%) were confirmed to harbor at least one pAmpC-encoding gene by PCR. These 57 strains were acquired by 49 travelers.

The global acquisition rate of pAmpC-E in the cohort of travelers was 8.5% (49/574), ranging from 5.1% (10/195), 7.6% (14/184) to 12.8% (25/195) for those having traveled in Africa, Latin America or Asia, respectively (S2 Table). Among travelers to popular destinations (>10 study participants) who visited only one country, pAmpC-E acquisition was most frequent among those who visited Peru (21.9%), India (21.4%), Vietnam (20%) and Indonesia (17.6%). Among the 49 travelers who acquired at least one pAmpC-E, 34 (69%) acquired another MRE (ESBL-E for 33/34 subjects, ESBL-E plus carbapenemase producing Enterobacteriaceae [CPE] for the last one). Thus, only 15 travelers acquired only pAmpC-E during their travels (7 from America, 6 from Asia and 2 from Africa) (S2 Table).

### Variables associated with pAmpC-E acquisition

A univariate multinomial logistic regression univariate was performed. Factors significantly associated with the acquisition of pAmpC-E compared to travelers who had not acquired any MRE, were diarrhea during travel (relative odds ratio [r-OR] 2.06; 95% confidence interval [IC] 1.12–3.81; P = 0.021), antibiotic use during travel (r-OR, 2.74; 95%CI 1.06–7.06; P = 0.037) and the geographic area visited, Asia being most at risk (Table 1). Risk factors associated with the acquisition of pAmpC-E are also those associated with the acquisition of ESBL-E.

**Table 1:** Univariate multinomial logistic regression analysis in travelers with pAmpC producing Enterobacteriaceae, ESBL producing Enterobacteriaceae or without any MRE acquisition.

	Total	Travelers with pAmpC-E acquisition	Travelers with only ESBL-E acquisition	Travelers without any MRE acquisition	pAmpC-E group compared to no acquisition group (Univariate Analysis)		only ESBL group compared to no acquisition group (Univariate Analysis)	
Variable		No. (%)	No. (%)	No. (%)	Relative Odds Ratio (95% CI)	p-value	Relatif Odds Ratio (95% CI)	p-value
**Age**								
Total	574	49	243	282				
[18.34]	334 (58)	30 (61)	153 (63)	151 (54)	1		1	
(34.49]	145 (25)	13 (27)	55 (23)	77 (27)	0.85 (0.42–1.72)	0.65	0.7 (0.47–1.07)	0.097
(49.64]	77 (13)	4 (8)	27 (11)	46 (16)	0.44 (0.15–1.31)	0.14	0.58 (0.34–0.98)	0.042
(64. ++]	18 (3)	2 (4)	8 (3)	8 (3)	0.78 (0.25–6.22)	0.78	0.99 (0.36–2.7)	0.98
**Sexe**								
Total								
Male (reference)	222 (39)	19 (39)	84 (35)	119 (42)	1		1	
Female	352 (61)	30 (61)	159 (65)	163 (58)	1.15 (0.62–2.15)	0.65	1.38 (0.97–1.97)	0.074
**Antibiotic use during travel (except doxycycline)**								
Total	566	49	238	279				
No (reference)	507 (89.6)	42 (85.7)	202 (84.9)	263 (94.3)	1		1	
Yes	59 (10.4)	7 (14.3)	36 (15.1)	16 (5.7)	2.74 (1.06–7.06)	0.037	2.93 (1.58–5.43)	0.00064
**Diarrhea during the travel**								
No (reference)	340 (60)	25 (51)	126 (52)	189 (68)	1		1	
Yes	228 (40)	24 (49)	116 (48)	88 (32)	2.06 (1.12–3.81)	0.021	1.98 (1.38–2.83)	0.00018
**Type of travel**								
Total	574	49	243	282				
All-inclusive resort	27 (5)	1 (2)	7 (3)	19 (7)	1		1	
Mix of all-inclusive resorts and organized tours	78 (14)	8 (16)	25 (10)	45 (16)	3.38 (0.39–28.93)	0.27	1.51 (0.56–4.08)	0.42
Family	142 (25)	9 (18)	54 (22)	79 (28)	2.17 (0.26–18.15)	0.48	1.86 (0.73–4.72)	0.19
Backpacking	200 (35)	18 (37)	105 (43)	77 (27)	4.44 (0.56–35.41)	0.16	3.7 (1.48–9.24)	0.005
Organized tour	127 (22)	13 (27)	52 (21)	62 (22)	3.99 (0.49–32.49)	0.20	2.28 (0.89–5.84)	0.087
**Visited region**								
Total	574	49	243	282				
Sub-Saharan Africa (reference)	195 (33.97)	10 (20.41)	83 (34.16)	102 (36.17)	1		1	
Latin America	195 (33.97)	25 (51.02)	116 (47.74)	54 (19.15)	1.13 (0.48–2.66)	0.77	0.43 (0.27–0.67)	0.00022
Asia	184 (32.06)	14 (28.57)	44 (18.11)	126 (44.68)	4.72 (2.11–10.55)	0.00016	2.64 (1.71–4.07)	0.000012
**Duration of travel (days)**								
Duration of travel. days. median (IQR)	21 [15:30]	22 [18:31]	22 [15.5:31]	19 [13.2:27.7]	1 (0.99–1.01)	0.69	1 (1–1.01)	0.67

### Description of the pAmpC-E

In all, pAmpC-E represented 10.6% (57/537) of all MRE isolated in the VOYAG-R study, and 14.5%, 10.6% and 7.4% of those acquired respectively in Latin America, Asia and Africa. Among the 57 pAmpC-E, 8 (14%) co-produced an ESBL.

The pAmpC strains were highly resistant to fluoroquinolones (77.2%), to tetracycline (75.4%) and to co-trimoxazole (52.6%). The molecules which remain the most often actives were aminoglycosides (5.3 and 19.3% resistance rate for amikacin and gentamicin, respectively) and fosfomycin (10.5%).

CMY-2-like enzymes were highly predominant (96.5%, 55/57) and all isolated in *E*. *coli*. The latter were mostly from the phylogroup A (18.2%), B1 (25.5%) and C (16.4%) and to a lesser extent to the extra-intestinal virulent B2 (7.3%), D (16.4%) and F (12.7%) phylogroups. Two *E*. *coli* (3.5%) belonged to the ST131-O25b clonal complex.

The two remaining were DHA enzymes (3.5%, 2/57) detected in *Klebsiella pneumoniae*.

In the 55 strains producing a CMY-2-like, the most prevalent were CMY-2 (n = 40), followed by CMY-42 (n = 12) and CMY-6 (n = 1). We also observed two new variants named, CMY-2m, variant of CMY-2 with a substitution of glycin 234 for arginin (G234R) and CMY-42m, variant of CMY-42 with a substitution of serine 309 for asparagine (S309N) (S1 Fig).

The CMY-2 producing strains were acquired in the three geographic areas whereas the others variants (CMY-42, CMY-6 and the two news variants), except for one CMY-42, were all acquired in Asia (Table 2).

**Table 2:** Enzyme acquired depending on the geographic area visited.

	Total	Sub-Saharian Africa	Latin America	Asia
	n %	n (%)	n (%)	n (%)
**CMY-2 like**	**55 (96.5)**	**9 (90.0)**	**15 (93.8)**	**31 (100)**
CMY-2	40 (70.2)	8 (80.0)	15 (93.8)	17 (54.8)
CMY-6	1 (1.8)	0 (0.0)	0 (0.0)	1 (3.2)
CMY-2m	1 (1.8)	0 (0.0)	0 (0.0)	1 (3.2)
CMY-42	12 (21.1)	1 (10.0)	0 (0.0)	11 (35.5)
CMY-42m	1 (1.8)	0 (0.0)	0 (0.0)	1 (3.2)
**DHA**	**2 (3.5)**	**1 (10.0)**	**1 (6.3)**	**0 (0.0)**
**Total**	**57 (100)**	**10 (100)**	**16 (100)**	**31 (100)**

**Fig 1:**
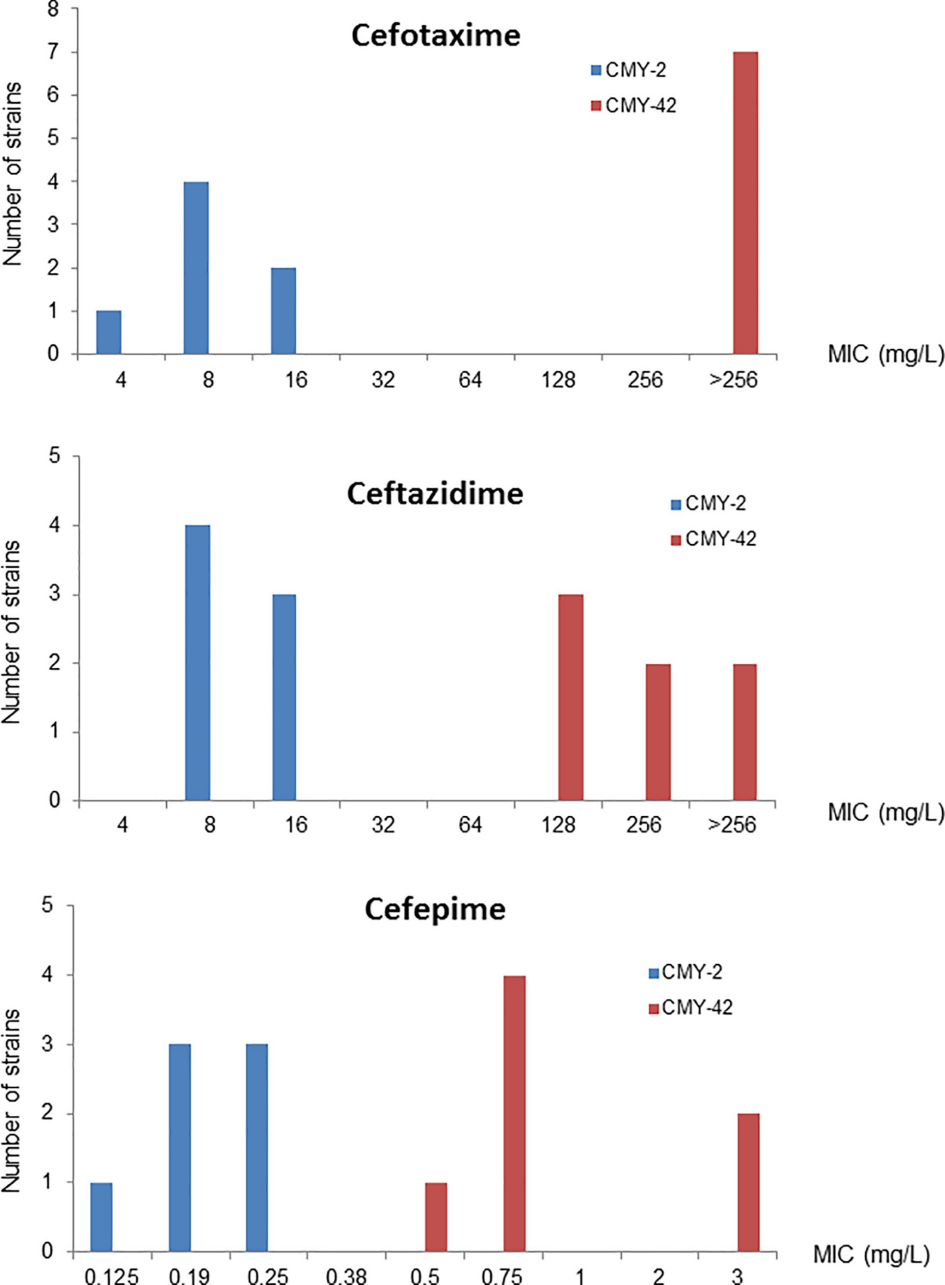
MICs (mg/L) of cefotaxime, ceftazidime and cefepime determined on the seven *E*. *coli* producing only a CMI-42 enzyme (without ESBL) and on seven, randomly chosen *E*. *coli* producing only a CMY-2 enzyme.

Cefotaxime, ceftazidime and cefepime MICs were performed on the seven *E*. *coli* producing only a CMI-42 enzyme (without ESBL) and on seven, randomly chosen, *E*. *coli* producing only a CMY-2 enzyme. The median MICs of cefotaxime (>256 *vs* 8 mg/L), ceftazidime (192 *vs* 8 mg/L) and cefepime (0.75 *vs* 0.19 mg/L) were much higher for CMY-42 producing strains than for CMY-2 ones (Fig 1)

CMY-2 encoding genes were mostly associated with IncI1 plasmid (45.2%) or IncF-IncI (7.5%), the others being IncF (10%), IncA/C (5.0%) and IncR (2.5%). CMY-42 genes were always associated with Incl1 plasmid with or without IncF (Table 3).

**Table 3:** Type of plasmid according to the pAmpC enzyme.

	CMY-2 like producing strains			DHA producing strains
Plasmid type	CMY-2	CMY-42	Others^a^	
	n (%)	n (%)	n (%)	n (%)
**IncI1**	17 (42.5)	10 (83.3)	2 (66.7)	-
**IncF**	4 (10.0)	-	-	-
**IncFIIk**	-	-	-	1 (50)
**IncA/C**	2 (5.0)	-	1 (33.3)	-
**IncR**	1 (2.5)	-	-	-
**IncF-IncI1**	3 (7.5)	2 (16.7)	-	-
**IncI1-IncR**	1 (2.5)	-	-	-
**IncI-IncN**	1 (2.5)	-	-	-
**Untypable**	11 (27.5)	-	-	1 (50)

^a^ CMY-6, CMY-42m et CMY-2m

### Follow-up of pAmpC carriers after return

**Fig 2:**
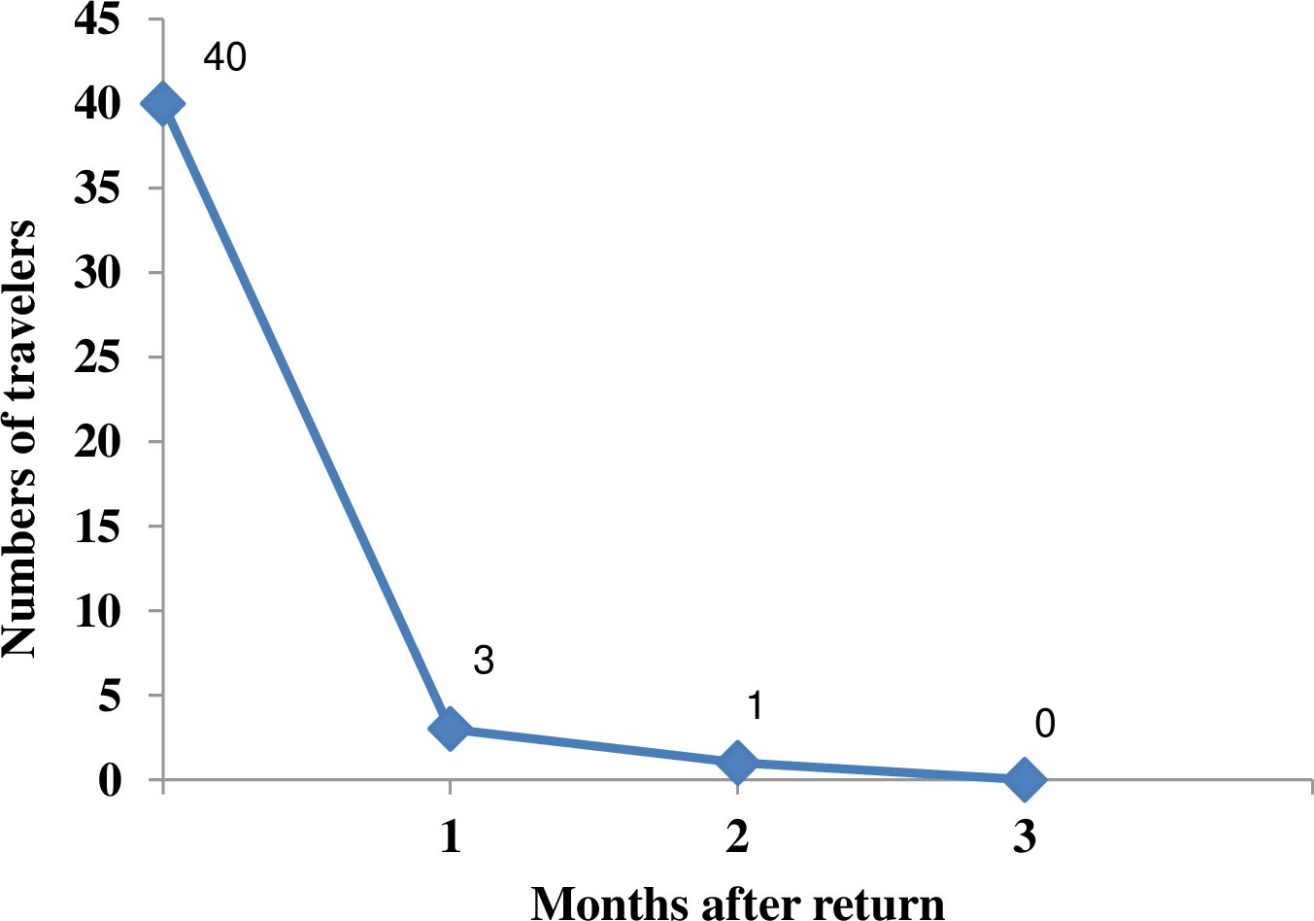
Follow-up of carriage of pAmpC-E after return.

Among the 49 travelers who acquired a pAmpC-E during travel, 40 provided a stool sample one month after their return, which were pAmpC-E positive in 3 cases (7.5%). Two months after their return, only one of the 3 subjects was still carrying a pAmpC. None was still positive three months after the return (Fig 2). The rep-PCR performed on strains isolated at M1 and M2 showed that, in a given traveler, strains were not distinguishable (S2 Fig). The three persistent strains were *E*. *coli* belonged to phylogroup D (n = 2) and B (n = 1).

## Discussion

We observed that the rate of pAmpC-E acquisition was 8.5% among travelers returning from tropical regions, and that it varied according to the visited country. These results are far from negligible even if they are well below the acquisition rate of ESBL-E (48%) observed in the VOYAG-R study [4].

Only two previous studies reported the acquisition rates of pAmpC-E in travelers with rates being lower than in the VOYAG-R study: 5.3% in Swedish travelers and 3.9% in Australian travelers [16,17]. This can be explained by the fact that we (i) included only travelers to tropical areas, (ii) used fresh stools and not rectal swabs and (iii) plated the feces onto a biplate agar containing cefotaxime and ceftazidime with an additional enrichment step for detection [4]. Unlike the ChromID ESBL agar medium, this biplate agar was not selective for ESBL-E and allowed also the growth of pAmpC-E not co-producing an ESBL.

As showed for ESBL-E, the acquisition rate of pAmpC-E was higher in Asia (12.8%), but, we did not observed differences between acquisition rate in Latin America (7.7%) and in Africa (5.1%). Interestingly, a very high acquisition rate was observed in Peru (22%) which represents half of acquired strains of pAmpC-E in Latin America. However, no data are available on the prevalence of pAmpC-E in Peru neither in hospital, nor in community setting.

Indeed, very few data report the prevalence of carriage of pAmpC-E in the community setting. In Europe, studies performed in the Netherland, in Switzerland and in Spain, estimated from 0.3 to 1.3% the digestive colonization with pAmpC [6,9,18]. In tropical areas, no data on prevalence of pAmpC-E carriage in the community setting are available. However, in clinical strains, prevalence of pAmpC-E varied from 0.1 to 3% whatever the region except for Asia were prevalence ranged from 0.1 to 45% depending of countries, India, South Korea and China having the highest rates [19–22]. Moreover, several studies reported an increase in the prevalence of pAmpC [10,19,23]. In addition to the travel destination, antibiotic exposure and occurrence of diarrhea were identified as risk factors associated with the acquisition of pAmpC-E. In most cases, patients who have acquired at least one pAmpC-E have also acquired an ESBL-E or a strain coproducing a pAmpC and an ESBL, so it is therefore logical to observe that risk factors associated with the acquisition of pAmpC-E were also present with the acquisition of ESBL-E. However, some risk factors such as the type of travel and age between 49 and 64 years associated with ESBL-E acquisitions were not found with pAmpC, probably due to the low number of pAmpc-E acquisitions and therefore a lack of statistical analysis power.

The duration of carriage of pAmpC-E after return seems shorter than that of ESBL-E, as only 7.5% of travelers who acquired a pAmpC-E were still carriers one month after return and none were carriers three months after. Indeed, 34% and 10% of travelers who acquired an ESBL-E were still carriers one month and three months respectively after their return [4]. Interestingly, while most pAmpC-*E*. *coli* strains belonged to non-virulent commensal phylogroups (A, B1 and C), strains still present one or two months after return belonged all to extra-intestinal virulent phylogroups B2 and D, suggesting that they possess characteristics allowing them to colonize more efficiently the gastro-intestinal tract [24].

Last, the characterization of the pAmpC enzymes confirms the global dissemination of CMY-2 like enzymes and their predominance in pAmpC producing *E*. *coli* [9,25]. Only two strains were not CMY-like, and were DHA producing *K*. *pneumoniae*. CMY-2 variant represented almost half of all isolated strains and were isolated worldwide. But, the second most important result of this study is that, surprisingly, CMY-42, a poorly documented variant, seems to have spread in Asia and mostly in India. It represents 40% of CMY variant isolated in travelers from Asia. This variant had previously been isolated sporadically in few patients from China, India, Egypt and Spain [26–29] and also in urban water in India, confirming its presence in the environment of this country [30]. Very recently, a study reported a prevalence of 18% of CMY-42 in clinical samples from a tertiary referral hospital of India [31]. By comparing nucleotide sequence of CMY-42 to CMY-2, the difference of two amino-acids located in the omega loop of the enzyme confers an increasing resistance to third generation cephalosporins and to cefepime, usually spared by pAmpC [32]. Thus, the broader spectrum of this enzyme could be an explanation of its increasing rate. The detection of two new variants on 57 acquired strains probably reflects the constant evolution of bacteria in environments with high selection pressure, that it could be often the case in India and also the lack of current interest and of knowledge on pAmpC-E.

In the literature, the main part of *bla*_CMY-2-like_ genes has been found on IncA/C or IncI1 plasmids [6,33]. In our study, most of *bla*CMY-2 and all *bla*CMY-42 genes were associated with IncI plasmid. In contrast to *bla*CTX-M genes, *bla*CMYs are rarely carried by IncF plasmids. However, IncF plasmids have been shown to play a key role in the spread of the CTX-M enzyme [34]. IncI plasmids may have lower disseminating properties which may, in part, explain the lower spread of pAmpC-E compared to ESBL-E. However, CMY producing Enterobacteriaceae seems to increase worldwide and the description of CMY genes in the successful clone of ST131 *E*. *coli* may raise concerns about the risk of widespread spread in the future [22].

However, our study has some limitations since pAmpC genes have only been investigated in species, except *E*. *coli*, which did not harbor chromosomal *ampC* and since *bla*_ACT/MIR_ and *bla*_FOX_ have not been screened. Although these species and genes have poorly been associated in the literature with community carriage, this may have underestimated the rate of pAmpC-E acquisition among our travelers.

In conclusion, the pAmpC-E acquisition rate during a travel to tropical areas was much lower than the ESBL-E rate, but it shall not be considered as negligible. The increasing detection of pAmpC enzyme in humans and especially in the community requires attention to their evolution.

## Supporting information

S1 Table Primers used for detection of p-AmpC genes. (PDF)

S2 Table Acquisition rate of pAmpC-E and other type of MRE depending on the geographic area visited. (PDF)

S1 Fig Nucleotide (A) and protein (B) sequences of the isolated variants of CMY-2like genes.
(TIF)

S2 Fig Comparison by Rep-PCR of the strains isolated at return of travelers, one (M1) and two (M2) months later. (TIF)
