# Supplementary material for: Acquisition of plasmid-mediated cephalosporinase producing Enterobacteriaceae after a travel to the tropics
Source: PLoS One. 2018 Dec 18;13(12):e0206909. doi: 10.1371/journal.pone.0206909 (PMC6298645; doi:10.1371/journal.pone.0206909)
Supplement: S1 Table — (PDF) [file pone.0206909.s001.pdf]

S1 Table. Primers used for detection of p-AmpC genes

Target	Cluster	Primers	Sequence of primers
***bla*$_{\mathbf{CMY\text{-}2\ like}}$**	*C. freundii*	CIT F	5’-GGCGGGTTTACCTCAACGGC-3’
		CIT R	5’-TGCTGCTGACAGCCTCTTTCTC-3’
***bla*$_{\mathbf{DHA}}$**	*M. morganii*	DHA F	5’-CATTAAACCGCTGATGGCACAG-3’
		DHA R	5’-GCCAGAATCACAATCGCCACC-3’
***bla*$_{\mathbf{MOX}}$**	*Aeromonas*	MOX F	5’-CAACAACGACAATCCATCCTGTG-3’
		MOX R	5’-TTCGGCACATTGACATAGGTG-3’
***bla*$_{\mathbf{ACC}}$**	*H. alvei*	ACC F	5'-AAGTGGGTTCGCTGAGTAAA-3’
		ACC R	5'-CACCGAGCCGTTAGTTGAT-3’
