# Supplementary material for: Acquisition of plasmid-mediated cephalosporinase producing Enterobacteriaceae after a travel to the tropics
Source: PLoS One. 2018 Dec 18;13(12):e0206909. doi: 10.1371/journal.pone.0206909 (PMC6298645; doi:10.1371/journal.pone.0206909)
Supplement: S2 Table — (PDF) [file pone.0206909.s002.pdf]

S2 Table. Acquisition rate of pAmpC-E and other type of MRE depending on the geographic area visited

	**Total (n=574)**	**Sub-Saharian Africa (n=195)**	**Latin America (n=183)**	**Asia (n=196)**
	n (%)	n (%)	n (%)	n (%)
**pAmpC-E**[a]	**49 (8.5)**	**10 (5.1)**	**14 (7.7)**	**25 (12.8)**
E-ESBL [b, e]	277 (48.3)	91 (46.7)	50 (27.3)	136 (69.4)
CPE [c, e]	3 (0.5)	0 (0)	0 (0)	3 (1.5)
Total MRE[d, e]	292 (50.9)	93 (47.7)	57 (31.1)	142 (72.4)

[a] pAmpC-E: plasmid borne AmpC producing Enterobacteriaceae
[b] ESBL-E: extended spectrum beta-lactamase producing Enterobacteriaceae
[c] CPE: carbapenemase producing Enterobacteriaceae
[d] MRE: multi-drug resistant Enterobacteriaceae
[e] Results from the VOYAG-R study [4]. Travellers may have acquired several type of MRE
