# Supplementary figures and images for: Acquisition of plasmid-mediated cephalosporinase producing Enterobacteriaceae after a travel to the tropics
Source: PLoS One. 2018 Dec 18;13(12):e0206909. doi: 10.1371/journal.pone.0206909 (PMC6298645; doi:10.1371/journal.pone.0206909)

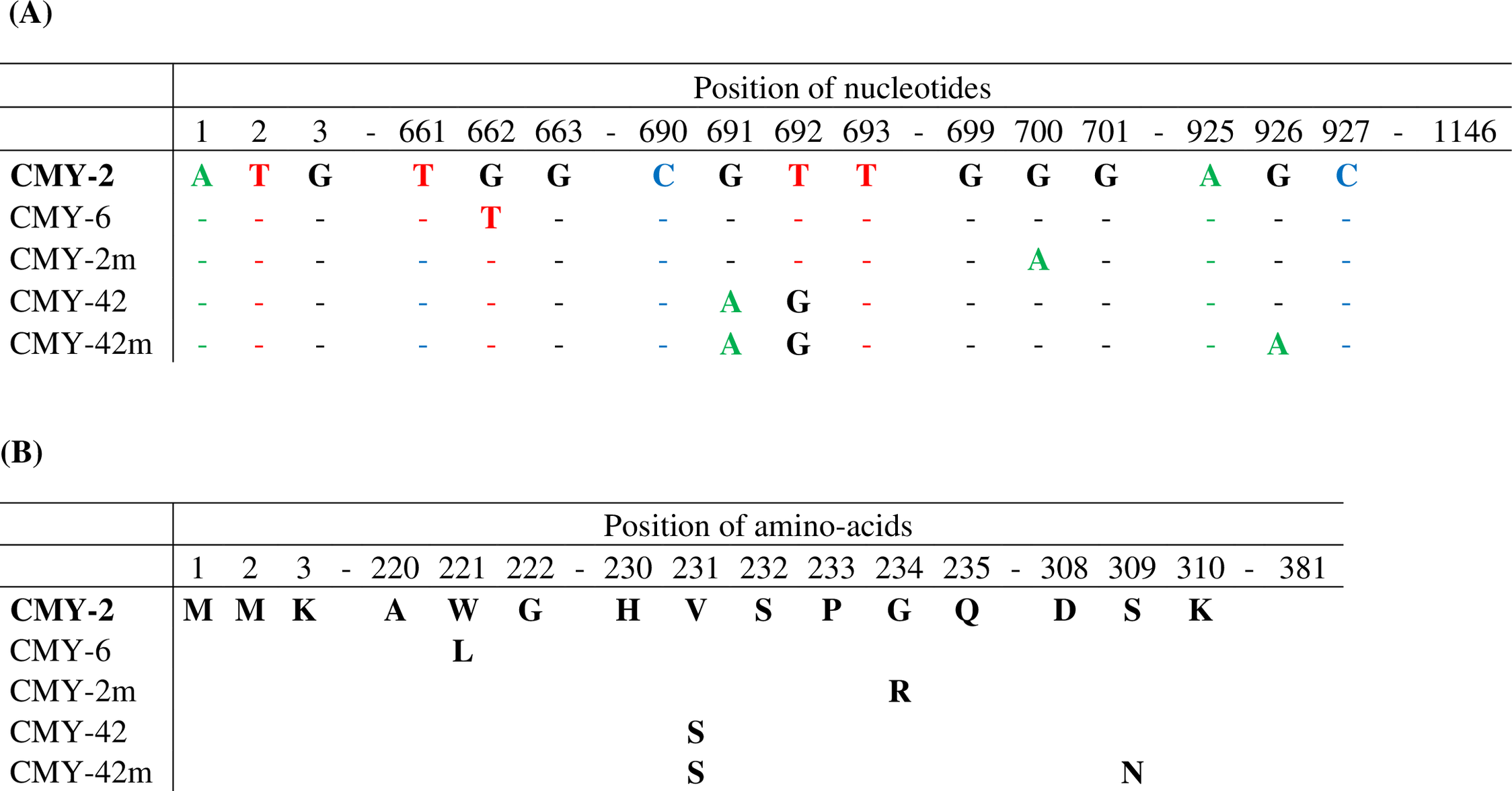

Supplement: S1 Fig — Nucleotide (A) and protein (B) sequences of the isolated variants of CMY-2like genes. (TIF) [file pone.0206909.s003.tif]

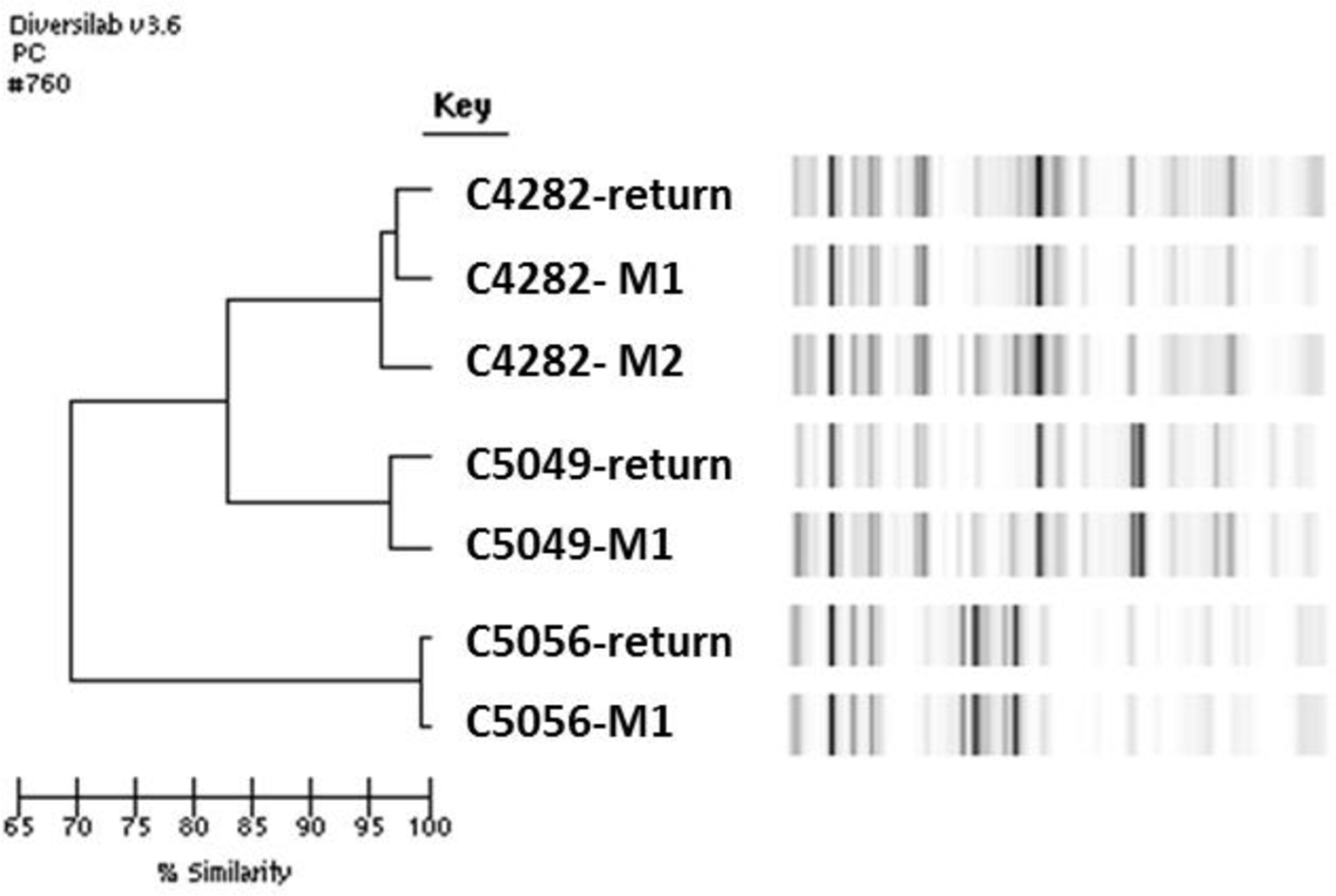

Supplement: S2 Fig — (TIF) [file pone.0206909.s004.tif]
